# Supplementary figures and images for: Effects of Degradation on Microbial Communities of an Amazonian Mangrove
Source: Microorganisms. 2023 May 25;11(6):1389. doi: 10.3390/microorganisms11061389 (PMC10301220; doi:10.3390/microorganisms11061389)

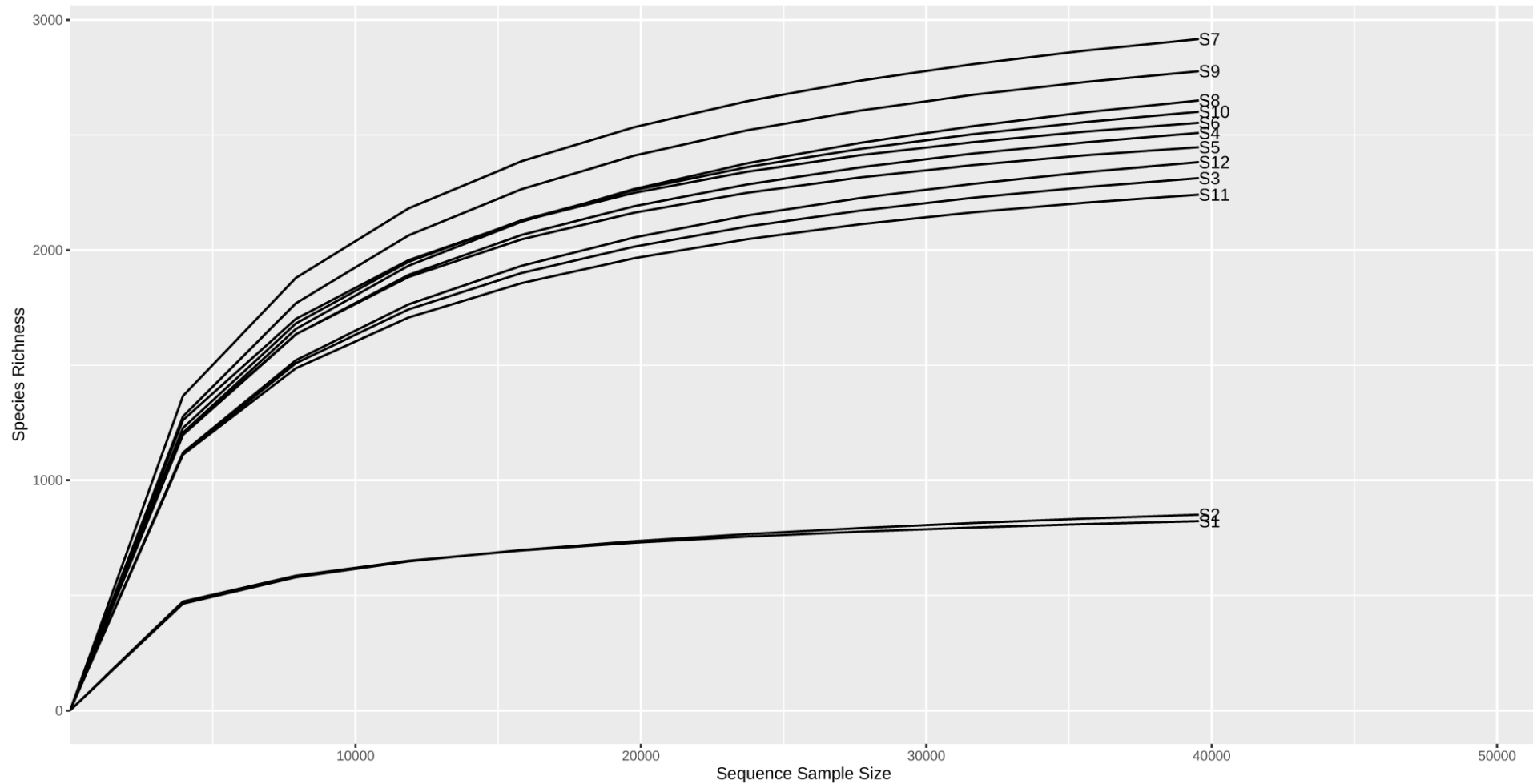

Figure S1: Rarefaction curve

Supplement: Supplementary file 1 [file microorganisms-11-01389-s001.zip › microorganisms-2329836-supplementary.pdf]
